# Reviewed article: Gonçalves CFG, Madeiro AP. Association between physical activity and the risk of prenatal depression in adolescents in Teresina, Piauí, Brazil: cross-sectional study, 2023-2024. Epidemiol Serv Saude. 2025:34;e20240789

**DOI:** 10.1590/S2237-96222025v34e20240789.a

**Published:** 2025-06-20

**Authors:** Luzimere do Nascimento

**Affiliations:** ENSP, Doutorado em Saúde Pública e Meio Ambiente

**Table:** 

Rating	Excellent	Good	Average	Below average	Poor
Interest	✓				
Quality		✓			
Originality	✓				
Overall		✓			

**Table:** 

Questionnaire	Yes	No	Not applicable
Does the manuscript contain new and significant information to justify publication?	✓		
Does the Abstract (Summary) clearly and accurately describe the content of the article?	✓		
Is the problem significant and concisely stated?	✓		
Are the methods described comprehensively?	✓		
Are the interpretations and conclusions justified by the results?	✓		
Is adequate reference made to other work in the field?	✓		

## Manuscript Structure

Length of article is: Adequate

Number of tables is: Adequate

Number of figures is: Adequate

Please state any conflict(s) of interest that you have in relation to the review of this paper (state “none” if this is not applicable): None

## Comments

Parabéns pela pesquisa e contribuição. 

